# Coordination and Metal Ion–Catalyzed Oxidation of a Mutated Prion Protein Fragment

**DOI:** 10.1155/bca/8800140

**Published:** 2026-10-21

**Authors:** Antal Galvácsi, Roland Császár, Lajos Nagy, Monika Katarzyna Lesiów, Nóra V. May, Csilla Kállay

**Affiliations:** ^1^ Department of Inorganic and Analytical Chemistry University of Debrecen H-4032 Debrecen Hungary unideb.hu; ^2^ Department of Applied Chemistry University of Debrecen H-4032 Debrecen Hungary unideb.hu; ^3^ Faculty of Chemistry University of Wrocław Fryderyka Joliot-Curie 14, 50-383 Wrocław Poland uni.wroc.pl; ^4^ Centre for Structural Science Research Centre for Natural Sciences Hungarian Research Network (HUN-REN) Magyar Tudósok Körútja 2, H-1117 Budapest Hungary ttk.hu

## Abstract

Metal ion coordination and metal‐catalyzed oxidative processes play a key role in the pathology of neurodegenerative diseases involving prion protein peptide. In this work, the complex formation and copper(II)‐catalyzed oxidation of a prion protein–derived model peptide, H_2_N‐GTHSAKHA‐NH_2_, were investigated. Complex formation with Cu(II), Ni(II), and Zn(II) ions was studied by pH‐potentiometry, UV–vis, EPR, and circular dichroism spectroscopy. The results demonstrate the formation of mono‐ and dinuclear complexes, with a strong preference for albumin‐like N‐terminal coordination involving the amino group, deprotonated amide nitrogen atoms, and histidine imidazole donors. Copper(II) dominates at the N‐terminal site also in mixed‐metal systems, whereas nickel(II) and zinc(II) bind only to secondary C‐terminal sites. HPLC and LC–MS studies revealed that treatment with the Cu/H_2_O_2_ system selectively oxidized the C‐terminal histidine residue, yielding 2‐oxo‐histidine. The effects of potential antioxidants on this oxidation process were also investigated. The addition of ascorbic acid significantly enhanced reactive oxygen species generation, resulting in increased peptide oxidation and DNA degradation, whereas the salan‐based Salpyran ligand inhibited oxidation.

## 1. Introduction

The interaction of metal ions with prion protein peptide is directly related to the development of various neurodegenerative diseases. Neurodegenerative disorders encompass a variety of pathological conditions such as protein aggregation and oxidative stress, both of which are associated with the involvement of metal ions, especially copper(II) ions. The role of metal ions in the development of degenerative diseases has become a major focus of bioinorganic research. A deep understanding of the interaction of metal ions with this protein requires the detailed studies of coordination and oxidation processes. The transition metal ion complexes of prion protein peptide fragments were studied in detail.

Human prion protein (Hu–PrP) is a membrane‐anchored cell surface glycoprotein containing 231 amino acid residues. From a coordination chemistry point of view, the most important structural feature of prion protein is its high histidine content. Altogether, the protein contains 10 histidine residues, which are well separated from each other. Therefore, all of them are effective and independent metal binding sites. Several species with different stoichiometries, various coordination isomers, macrochelates, and polynuclear complexes can be formed with transition metal ions [1–4].

Research on the oxidative damage of prion proteins dates back more than 20 years [5], and our research group has contributed to this field through systematic studies of the oxidation of Hu–PrP fragments. Oxidation of the prion protein predominantly affects its methionine and histidine residues. [6–8]. The position of methionine and the effects of adding ascorbic acid and other compounds were also investigated. It has been established that methionine oxidation invariably occurs, independent of the methionine residue’s position. It has been demonstrated that the presence of methionine residues protects peptides from fragmentation, but the methionine residue is oxidized to methionine sulfoxide. In turn, peptides lacking methionine undergo degradation [9].

The histidine residues at different positions of the peptide chain exhibit varying sensitivities to oxidation, and their order of reactivity has been established for a model peptide of the Hu–PrP [10]. Interestingly, this is not directly related to the metal‐binding affinity of the binding sites, suggesting that steric effects play a decisive role. Additionally, the impact of ascorbic acid on the metal‐catalyzed oxidation of a human PrP model peptide was investigated, demonstrating the ambivalent role of ascorbic acid in this process [11].

Besides ascorbic acid, the effect of different types of antioxidants and metal chelators [12] was studied in different research groups. These molecules have proven to be a promising approach for slowing AD progression by inhibiting oxidative stress. In our experience, metal‐protein attenuating compounds (MPACs) such as aroylhydrazones and salan‐based ligands [13–15] also effectively prevented the oxidation of methionine and histidine.

The N‐terminus of the previously studied prion protein fragments was protected by acetylation. In contrast, the studied prion protein–derived segment contains a free amino group at the N‐terminus, which significantly changes the coordination mode of the peptide. This modification creates ATCUN‐like metal‐binding motifs due to the presence of the histidine residue in the third position. The studied H_2_N‐GTHSAKHA‐NH_2_ model peptide contains the prion protein’s two metal‐binding sites (namely, His^96^ and His^111^) relatively close to each other, but like the amyloid beta, it contains an N‐terminal amino group, while the oxidation‐sensitive methionine residues, which protect the oxidation of histidine, are replaced by alanine residues.

The aim of this study is to investigate the effect of the coordination chemistry of this prion protein–derived peptide on the metal ion‐catalyzed oxidation. Its deviations from the native prion binding sites may help clarify how specific features of the metal coordination environment influence oxidative reactivity in the presence or absence of ascorbic acid and hydrogen peroxide. The formation of mono‐ and dinuclear, as well as mixed metal ion complexes, and the metal ion‐catalyzed oxidation of this peptide were studied. The addition of ascorbic acid to the complexes can result in the production of reactive oxygen species (ROS), which may lead to peptide oxidation. Therefore, we also investigated whether the studied compounds are capable of generating ROS and identified the formed species.

## 2. Materials and Methods

### 2.1. Materials

The H_2_N‐GTHSAKHA‐NH_2_ peptide was purchased from SynPeptide Co., Ltd. (Shanghai, China) with purity exceeding 99%. The concentration of the peptide stock solution was determined by pH‐potentiometric titration. Stock solutions of Cu(II), Ni(II), and Zn(II) were prepared from analytical‐grade CuCl_2_, NiCl_2_, and ZnCl_2_, and their concentrations were measured using gravimetry by oxinate precipitation. Potassium hydroxide (KOH) and potassium chloride (KCl), used for the pH‐potentiometric titrations, were obtained from Merck (Waltham, MA, USA).

### 2.2. pH Potentiometric Titrations

pH‐potentiometric titrations were performed in 3.00 mL samples containing the ligand at a concentration of 1.0 × 10^−3^ M. Metal‐to‐ligand molar ratios of 1:1 and 2:1 were used for the binary systems, while a 1:1:1 ratio was applied for the ternary systems, following previously described procedures [16].

The titrations were carried out using a carbonate‐free KOH stock solution of accurately known concentration as the titrant. pH measurements (pH 2.5–11.5) were performed with a MOLSPIN pH meter equipped with a Metrohm 6.0234.100 combined glass electrode, while the titrant was delivered by a computer‐controlled MOL‐ACS microburette. During the measurements, argon was continuously bubbled through the samples to exclude oxygen and carbon dioxide.

All measurements were performed at 298 K and at a constant ionic strength of 0.20 M (KCl). The measured pH values were converted to hydrogen ion concentrations according to the procedure of Irving et al. [17]. Protonation constants of the ligands and overall stability constants (log*β*_*p* *q* *r*_) of the metal complexes were determined using the SUPERQUAD [18] and PSEQUAD [19]) software packages based on equations (1) and (2).

(1) pM+qH+rL=MpHqLr,

(2) βpqr=MpHqLrMp·Hq·Lr.

### 2.3. Spectroscopic Measurements

UV–vis spectra of the metal complexes were recorded using a Hewlett Packard HP 8453 diode‐array spectrophotometer and a PerkinElmer Lambda 25 double‐beam spectrophotometer. Measurements were carried out at the same concentrations as those used in the pH‐potentiometric experiments.

Circular dichroism (CD) spectra were recorded on a JASCO J‐810 spectropolarimeter over the wavelength range of 200–800 nm using 0.100 cm and/or 1.00 cm path‐length quartz cells. The concentrations of the samples were identical to those used for the pH‐potentiometric measurements.

### 2.4. Oxidation of the Studied Peptide Fragment

Reaction mixtures containing 1.0 mM peptide were prepared at a 1:1 metal‐to‐ligand molar ratio and incubated at 25°C for various time periods in the presence of hydrogen peroxide at pH 7.4. The ligand‐to‐H_2_O_2_ molar ratio was maintained at 1:4. In selected experiments, Salpyran (2‐(((2‐((pyridin‐2‐ylmethyl)amino)ethyl)amino)methyl)phenol) was added in an equimolar amount relative to the metal ion, while ascorbic acid was applied in a 20‐fold molar excess (ligand‐to‐ascorbic acid ratio of 1:20). The reactions were initiated by the addition of freshly prepared 1% (v/v) hydrogen peroxide and quenched by adding a fivefold molar excess of Na_2_EDTA.

The reaction progress was monitored at selected time intervals by reversed‐phase high‐performance liquid chromatography (RP‐HPLC). Analytical RP‐HPLC was performed on a JASCO system equipped with an MD‐2010 Plus multiwavelength detector. Oxidation products were separated on a Teknokroma Europa Protein C18 column (250 × 4.6 mm, 300 Å, 5 μm) at a flow rate of 1.0 mL min^−1^ and detected at 222 nm. The mobile phases consisted of water (A) and acetonitrile (B), each containing 0.1% (v/v) trifluoroacetic acid (TFA). Elution was carried out using the following gradient of solvent A: 100% (0–5.5 min), linear decrease to 92% (5.5–7.0 min), linear increase to 100% (7.0–21.0 min) and 100% thereafter (21.0–30.0 min).

For the isolation of oxidation products, semipreparative RP‐HPLC was performed on a Jasco system equipped with a Jasco UV‐2077 Plus 4‐*λ* Intelligent UV/Vis detector using a Grace Vydac Protein and Peptide C18 218TP510 column (250 × 10 mm, 300 Å, 5 μm). The flow rate was maintained at 2.0 mL min^−1^, and peptide elution was monitored at 222 nm. The same mobile phases and gradient elution program as described for the analytical RP‐HPLC measurements were employed.

### 2.5. Mass Spectrometry

MS and MS/MS measurements were performed in positive ion mode using a MicroTOF‐Q Qq‐TOF mass spectrometer (Bruker Daltonik, Bremen, Germany) equipped with an electrospray ionization (ESI) source. The ESI source was operated at a spray voltage of 4 kV, with nitrogen used as the drying gas. The drying gas temperature was maintained at 200°C, and the gas flow rate was set to 4.0 L min^−1^ (8.0 L min^−1^ for LC–MS measurements). For MS/MS experiments, nitrogen was used as the collision gas at a collision cell pressure of 1.2 × 10^−2^ mbar. Precursor ions were isolated using a 5 m/z window, and MS/MS spectra were acquired with a digitizer operating at a sampling rate of 2 GHz. External mass calibration was performed using the exact masses of [(NaTFA)_n_ + Na]^+^ cluster ions generated by electrospraying a sodium trifluoroacetate (NaTFA) solution. Data were processed using DataAnalysis 3.4 software (Bruker). Samples were introduced directly into the ESI source by syringe infusion using a syringe pump (Cole‐Parmer Instrument Company, Vernon Hills, IL, USA) at a flow rate of 3 μL min^−1^. For LC–MS measurements, the flow rate was increased to 100 μL min^−1^.

### 2.6. EPR Measurements

Continuous‐wave (CW) EPR spectra were recorded on a BRUKER EleXsys E500 spectrometer operating at a microwave frequency of 9.85 GHz, with a microwave power of 13 mW, a modulation amplitude of 5 G, and a modulation frequency of 100 kHz. Cu(II)–NH_2_‐GTHSAKHA‐NH_2_ samples (1 mM) were prepared in aqueous solution at various pH values. Room‐temperature isotropic EPR spectra were recorded in capillary tubes. Frozen‐solution EPR spectra were obtained using quartz EPR tubes after adding 0.05 mL of methanol to each 0.20 mL sample to prevent water crystallization during freezing. The measurements were carried out at 77 K using a liquid nitrogen dewar.

All EPR spectra were simulated by the “epr” software [20, 21]. The isotropic spectra were simulated by considering the isotropic *g* value (*g*_0_) and the copper hyperfine coupling constant ($A_{0}^{\text{Cu}}$, *I*_Cu_ = 3/2). The linewidths were described by the relaxation parameters *α*, *β*, and *γ* according to the equation *σ*_MI_ = *α* + *β* *M*_*I*_ + *γ* $M_{I}^{2}$, where *M*_I_ is the magnetic quantum number of the paramagnetic copper ion.

The anisotropic EPR spectra were simulated assuming axial *g*‐ and *A*‐tensors (*I*_Cu_ = 3/2). Nitrogen superhyperfine splitting was not sufficiently resolved to allow inclusion of nitrogen hyperfine couplings in the simulations. The linewidths were described using orientation‐dependent *α*, β, and γ relaxation parameters according to the same equation, *σ*_MI_ = *α* + *β* *M*_*I*_ + *γ* $M_{I}^{2}$.

Because naturally abundant CuCl_2_ was used for sample preparation, both the isotropic and anisotropic spectra were simulated as the weighted sum of the contributions from ⁶³Cu and ⁶⁵Cu isotopes according to their natural abundances. Hyperfine and superhyperfine coupling constants, as well as the relaxation parameters, are reported in field units (*G*; 1 *G* = 10^−4^ T).

### 2.7. Ascorbate Consumption

Ascorbate consumption was monitored by UV–vis spectroscopy using a Cary 60 spectrophotometer (Agilent Technologies) at 25°C. The oxidation of ascorbate was followed by the decrease in its absorption band at 265 nm (*ε* = 1,45,000 M^−1^ cm^−1^) over 15 min [22]. The final concentrations were 10 μM Cu(II), 12 μM H_2_N‐GTHSAKHA‐NH_2_, 100 μM ascorbic acid, and 50 μM H_2_O_2_ in 10 mM phosphate‐buffered saline (PBS, pH 7.4).

### 2.8. •OH Detection

Hydroxyl radical (•OH) production by the Cu(II) complex (50 μM, metal‐to‐ligand ratio 1:1) in the presence of H_2_O_2_ (5 mM) was evaluated using the rhodamine 6G (R6G) degradation assay. •OH react with R6G, resulting in degradation of the dye and a corresponding decrease in its absorption band at 526 nm [23]. Samples were prepared in 10 mM PBS (pH 7.4). UV–vis spectra were recorded over the 400–600 nm range in 1 cm quartz cuvettes using a Cary 60 spectrophotometer (Agilent Technologies).

### 2.9. Gel Electrophoresis

The ability of the Cu(II):H_2_N‐GTHSAKHA‐NH_2_:H_2_O_2_ and Cu(II):H_2_N‐GTHSAKHA‐NH_2_:ascorbic acid: systems to generate ROS capable of inducing DNA damage was investigated using the pBR322 plasmid. Gel electrophoresis experiments were performed according to previously described procedures [24, 25]. Sodium azide (NaN_3_), dimethyl sulfoxide (DMSO), and potassium iodide (KI) were used as specific scavengers of singlet oxygen (^1^O_2_), •OH, and superoxide anion radicals (O_2_•^−^), respectively.

After incubation at 37°C for 1 h, the samples were mixed with bromophenol blue loading dye (30% glycerol) and loaded onto a 1% agarose gel containing ethidium bromide in TBE buffer (pH 7.4). The final concentration of each Cu(II)–peptide system was 50 μM with a 1:1 metal‐to‐ligand molar ratio. Electrophoresis was performed at a constant voltage of 120 V for 120 min. The gels were visualized and documented using a Digital Imaging System.

## 3. Results

### 3.1. Protonation Equilibria

The interaction of the peptide with copper(II), nickel(II), and zinc(II) ions was studied at 1:1 and 2:1 metal‐to‐ligand ratios. The deprotonation constants of the peptide and the stability constants of the formed complexes were determined by pH‐potentiometry. UV–visible and CD spectroscopy were used to identify the coordinating donor atoms and to determine the structures of the formed complexes. The peptide can be characterized by four deprotonation constants (Table 1). The deprotonation of the protonated imidazole nitrogen of the two histidyl side chains occurs in overlapping steps between pH 5.5 and pH 7.0; the two lowest constants cannot be assigned unambiguously to the individual histidyl side chains. The deprotonation of the N‐terminal ammonium group occurs around pH 8, and the highest value can be assigned to the deprotonation of the ε‐amino group of the lysyl side chain. These values are in good agreement with the deprotonation constants of the H_2_N‐GTHSMKHM‐NH_2_ peptide [21]. The peptide used for the comparison differs only in the replacement of two methionine residues by two alanine residues. The methionine side chains do not significantly affect the acid–base processes of the peptides, and therefore no significant difference in the constants is observed.

**TABLE 1:** pK values of H_2_N‐GTHSAKHA‐NH_2_ and the methionine‐containing H_2_N‐GTHSMKHM‐NH_2_ peptide used for comparison (I = 0.2 M KCl, *T* = 298 K, standard deviations are in parentheses).

	H_2_N‐GTHSAKHA‐NH_2_	H_2_N‐GTHSMKHM‐NH_2_ [26]
pK(Im_1_)	5.90 (4)	5.80
pK(Im_2_)	6.58 (4)	6.47
pK(NH_2_)	7.84 (3)	7.84
pK(ε‐NH_2_)	10.25 (2)	10.25

### 3.2. Copper(II), Nickel(II), and Zinc(II) Complexes

The complex formation reaction of the peptide with copper(II), nickel(II), and zinc(II) ions was studied at both 1:1 and 2:1 metal‐to‐ligand molar ratios. The stability of the formed complexes is summarized in Table 2. The distribution curves of the copper(II) ion‐containing systems are presented in Figure S1.

**TABLE 2:** Stability constants (logβ_pqr_) of the copper(II), nickel(II), and zinc(II) complexes of H_2_N‐GTHSAKHA‐NH_2_ and the methionine‐containing H_2_N‐GTHSMKHM‐NH_2_ peptide used for comparison (I = 0.2 M KCl, *T* = 298 K; standard deviations are in parentheses).

	H_2_N‐GTHSAKHA‐NH_2_			H_2_N‐GTHSMKHM‐NH_2_ [26]		
	Cu(II)	Ni(II)	Zn(II)	Cu(II)	Ni(II)	Zn(II)
[MLH]^3+^	19.78 (6)	15.66 (20)	14.12 (4)	19.60	—	14.38
[ML]^2+^	15.59 (2)	9.87 (9)	5.63 (14)	15.49	9.97	5.89
[MLH_–1_]^+^	9.85 (6)	3.29 (10)	−1.76 (3)	9.19	3.49	−1.58
[MLH_–2_]	−0.48 (9)	−7.39 (24)	−11.76 (3)	−1.19	−6.59	
[MLH_–3_]^−^	−11.72 (7)	−17.88 (13)		−12.51	−17.50	
[M_2_LH_–1_]^3+^			1.84 (10)			
[M_2_LH_–2_]^2+^	5.95 (48)	−2.59 (62)	−6.51 (17)	6.82	−2.29	
[M_2_LH_–3_]^+^	0.40 (6)			0.50		
[M_2_LH_–4_]	−7.15 (8)	−19.22 (16)		−7.33	−18.53	
[M_2_LH_–5_]^−^	−16.77 (5)	−28.24 (12)		−17.28	−27.89	

The studied peptide has several donor groups. In addition to the N‐terminal amino group, the two histidine side chains may also be included as anchor groups. But the primary metal ion binding site is the N‐terminal amino group, and complex formation is expected to start with the coordination of it [16]. The stoichiometry of the formed complexes is the same as in the case of the previously studied H_2_N‐GTHSMKHM‐NH_2_ peptide [26]. The same coordination mode is suggested according to the UV–vis and CD spectral parameters (Table 3). Only a slight increase in the stability of the species is observed, which can be explained by steric factors: the smaller size of alanine side groups compared to the replaced methionine ones. The complex formation process with the Cu(II) ion begins in the acidic pH range (around pH 4) at the N‐terminus. Because of the cooperative deprotonation of two amide nitrogen atoms in the equimolar solution, complexes with (NH_2_, N^–^, N^–^, and Im(N)) donor sets are formed, and they differ only in the protonation state of the lysyl side chain. It is supported by the UV–vis and CD spectra, where after the appearance of the d–d band, no shifts occur, only the intensity of this band increases (Figure S2). The EPR spectra also support the existence of species with the same coordination mode in the pH range 5.5 to 11.0. The experimental and simulated EPR spectra are shown in Figure S3. The EPR parameters in aqueous solution at 295 K were as follows: *g*_0_ = 2.097, *A*_0_ = 88 G. The anisotropic EPR parameters at 77 K were g_┴_ = 2.042, g_‖_ = 21,831, A_┴_ = 20 G, and A_‖_ = 199 G. The obtained parameters are in good agreement with those of species formed in an albumin‐like (NH_2_, N^–^, N^–^, and Im(N)) coordination environment [21].

**TABLE 3:** Spectroscopic data of the major species formed with copper(II) and nickel(II) ions with NH_2_‐GTHSAKHA‐NH_2_ and NH_2_‐GTHSMKHM‐NH_2_.

Species	Method	NH_2_‐GTHSAKHA‐NH_2_	NH_2_‐GTHSMKHM‐NH_2_ [26]
CuH_−1_L (and CuH_−2_L and CuH_−3_L)	UV–vis (*λ*_max_/*ε*, nm/dm^3^ mol^−1^ cm^−1^)	533/113	530/58
	CD (*λ*_max_/Δε, nm/dm^3^ mol^−1^ cm^−1^)	570/−0.93, 491/+0.65, 312/+1.90	570/−0.91, 480/+0.51, 311/+1.83

Cu_2_H_−5_L	UV–vis (*λ*_max_/*ε*, nm/dm^3^ mol^−1^ cm^−1^)	529 (119)	528/121
	CD (*λ*_max_/Δε, nm/dm^3^ mol^−1^ cm^−1^)	655/+1.13, 540/−1.13, 313/+281	655/+0.52, 550/−0.70, 315/+1.48

NiH_−1_L (and NiH_−2_L and NiH_−3_L)	UV–vis (*λ*_max_/*ε*, nm/dm^3^ mol^−1^ cm^−1^)	480/105 (sh), 426/158	480/95 (sh), 425/154
	CD (*λ*_max_/Δε, nm/dm^3^ mol^−1^ cm^−1^)	482/−2.50, 413/+1.79	480/−2.77, 412/+1.85

Ni_2_H_−5_L	UV–vis (*λ*_max_/*ε*, nm/dm^3^ mol^−1^ cm^−1^)	480/102 (sh), 432/146	480/119 (sh), 430/163
	CD (*λ*_max_/Δε, nm/dm^3^ mol^−1^ cm^−1^)	532/+0.65, 451/−0.65 (wide)	550/+0.22, 472/−1.03

The studied peptide can bind another copper(II) ion above pH 6. In this case, the His^111^ imidazole‐side chain is the anchoring group. Besides the C‐terminal Im(N) donor, dinuclear complexes are formed with the coordination of two or three deprotonated amide nitrogen atoms. The structure of the [Cu_2_LH_–5_]^–^ complex is presented in Scheme 1. The preference for the N‐terminal binding site is also evident from the comparison of the UV–vis and CD spectra of the equimolar (Figure S2) and 2:1 copper(II)‐peptide‐containing systems (Figure S4); these are in good agreement until pH 6, till the binding of the second copper(II) ion. This process is accompanied by a red shift in the UV–vis spectra and very significant changes in both the signs and intensities of the CD extrema. If the first copper(II) ion was coordinated to both binding sites in 50%–50% ratios, the coordination of the second copper(II) would result only in changes in the intensity of the spectra.

**Scheme 1:**
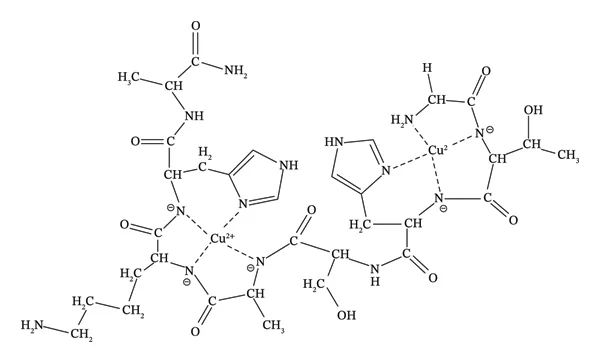
Structure of the [Cu_2_LH_–5_]^−^ species.

The complex formation process with the Ni(II) ion is similar to that observed with Cu(II); however, it begins at a higher pH, around 5.5. Mono‐ and dinuclear complexes form with the same stoichiometry and coordination modes.

At 1:1 and 2:1 nickel(II) ion to ligand ratios, the stoichiometry of the formed species and the coordination modes were the same as those observed for copper(II) ions; the first metal ion is coordinated by (NH_2_, N^–^, N^–^, and Im(N)) donor atoms, while the second metal ion is coordinated by (Im(N),N^–^,N^–^, and N^–^) donor atoms, where His7 is the anchor group. The coordination of the second nickel(II) ion occurs above pH 7 and is accompanied by significant changes, including the appearance of new bands in the CD spectra (Figure S5). The comparison of CD spectra at different metal ion to ligand ratios with those of model peptides supports these coordination modes (Figure 1).

**FIGURE 1:**
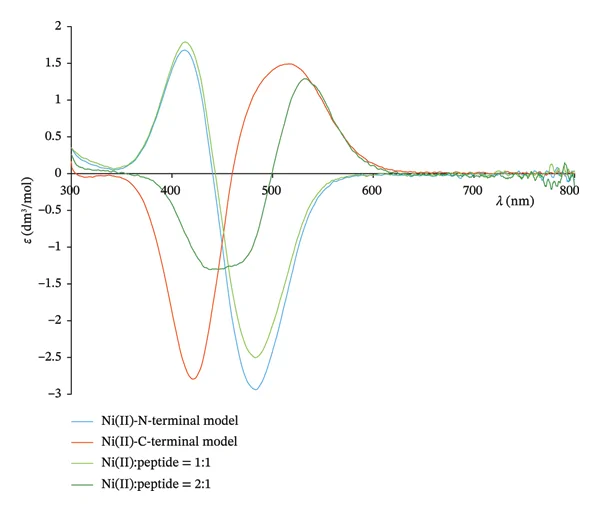
CD spectra of the Ni(II):H_2_N‐GTHSAKHA‐NH_2_ = 1:1 system and the peptide models mimicking the N‐terminal (H_2_N‐GTHS‐NH_2_) and C‐terminal binding sites (Ac‐SKPKATNAKHA‐NH_2_) of the Ni(II) ion.

The complex formation process with Zn(II) was also studied. This ligand is also able to bind zinc(II) ions even in the case of metal ion excess. No precipitate was observed in the solutions, and the UV–vis spectra did not indicate any precipitate formation. The equimolar solution can be described by [ZnLH]^3+^, [ZnL]^2+^, [ZnLH_–1_]^+^, and [ZnLH_–2_] species, while in the case of metal ion excess, [Zn_2_LH_–1_]^3+^ and [Zn_2_LH_–2_]^2+^ species are also formed. The deprotonation and coordination of the amide nitrogen atoms are not supported. In addition to the coordination of the N‐terminal amino group and the imidazole nitrogen atom, deprotonation of the coordinated water molecules occurs, leading to the formation of mixed hydroxo complexes.

### 3.3. Mixed Metal Complexes

#### 3.3.1. Cu(II)–Ni(II) System

Since the peptide is able to bind two equivalents of each studied metal ion, the formation of mixed metal complexes was also investigated. Stability constants were not determined, and no quantitative information was obtained for these systems. Conclusions were drawn solely from the comparison of the UV–vis and CD spectra.

The peptide is able to bind both metal ions in the copper–nickel system in the whole pH range. The spectral parameters of this system below pH 8 are in good agreement with those of the equimolar Cu(II)–H_2_N‐GTHSAKHA‐NH_2_ system (Table S1 and Table 3). Based on this fact, the coordination of the Cu(II) ions begins at the N‐terminus, similar to the Cu(II):ligand = 1:1 system. Above pH 8, the Ni(II) ion coordinates at the C‐terminus, similar to the second equivalent Ni(II) ion in the Ni(II):ligand = 2:1 system. These coordination modes are also confirmed by the comparison of the various CD spectra (Figure 2). This clearly demonstrates the N‐terminal coordination of copper(II) ions and the C‐terminal coordination of nickel(II) ions. The H_2_N‐GTHS‐NH_2_ peptide [26] mimics the N‐terminal site of the peptide, while Ac‐SKPKTNAKHA‐NH_2_ peptide [9] mimics the C‐terminal binding site. The coordination of nickel(II) ions at the N‐terminal would result in a significant decrease in the negative Cotton effect around 420 nm and a significant decrease in the positive Cotton effect around 500 nm. The sum of CD spectra of the systems (red solid line) in which the Cu(II) ions are coordinated at the N‐terminal (dark blue solid line) and Ni(II) ions are coordinated at the C‐terminal (dark green solid line) are in good agreement with the spectra of the measured mixed metal ion system (black solid line).

**FIGURE 2:**
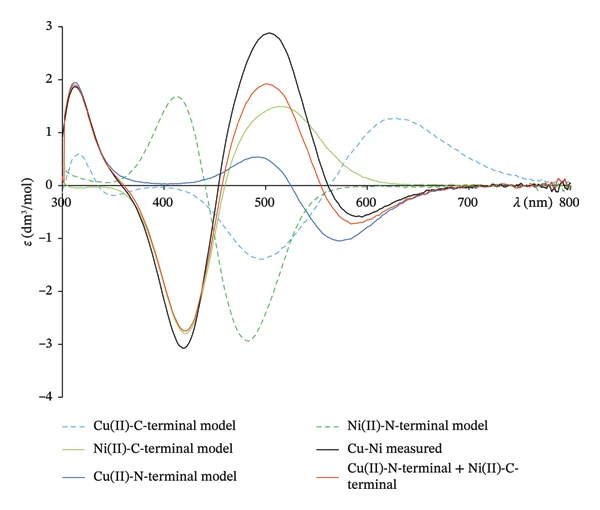
CD spectra of the Cu(II)–Ni(II):H_2_N‐GTHSAKHA‐NH_2_ = 1:1:1 system and the peptide models mimicking the Cu(II) and Ni(II) ion binding sites of it (the peptides mimicking the N‐ and C‐terminal binding sites are H_2_N‐GTHS‐NH_2_ [26] and Ac‐SKPKTNAKHA‐NH_2_ [9], respectively).

#### 3.3.2. Zn(II)‐Containing Mixed Metal Ion Systems

Both the Cu(II):Zn(II):H_2_N‐GTHSAKHA‐NH_2_ = 1:1:1 and Ni(II):Zn(II):H_2_N‐GTHSAKHA‐NH_2_ = 1:1:1 systems were studied by pH potentiometry and UV–vis and CD spectroscopy. The increase in absorbance in the UV–vis spectra (Figure S6) reveals that precipitation occurs around pH 8–10 in the zinc(II) ion‐containing mixed metal ion systems. This means that the peptide is not able to bind zinc ions in the presence of either copper(II) or nickel(II) ions in this pH range. However, the decrease in absorbance in the UV–vis spectra around pH 11 indicates coordination of zinc(II) ions. The absorption maxima of the mixed ligand systems (Table S1) are in good agreement with the binary systems (Table 3); 532 nm in the Cu(II):Zn(II):H_2_N‐GTHSAKHA‐NH_2_ = 1:1:1 and 427 nm in the Ni(II):Zn(II):H_2_N‐GTHSAKHA‐NH_2_ = 1:1:1 system. This indicates that the presence of zinc ions does not affect the coordination of either copper(II) or nickel(II) ions. Both metal ions are bonded in the same way to the N‐terminus as they do in the binary systems, and zinc(II) ions only occupy the free binding sites, as is demonstrated by the comparison of the CD spectra. There is no significant shift in the CD parameters, as can be seen by the comparison of the data in Table 3 and Table S1.

### 3.4. Copper(II)‐Catalyzed Oxidation of H_2_N‐GTHSAKHA‐NH_2_

The H_2_N‐GTHSAKHA‐NH_2_ peptide was oxidized with the Cu(II):L:H_2_O_2_ = 1:1:4 and Cu(II):L:H_2_O_2_:ascorbic acid = 1:1:4:20 systems at pH 7.4, where almost exclusively the (NH_2_, N^–^, N^–^, and Im(N)) coordinated [CuLH_–1_]^+^ species are present (Figure S1a). The peptide contains two histidine residues, which are the only potential targets for metal‐catalyzed oxidation. Based on literature, the formation of 2‐oxo‐histidine derivatives can be supposed in the studied systems [27–31].

The HPLC chromatogram of the peptide and the products formed in the Cu(II): Cu(II):H_2_N‐GTHSAKHA‐NH_2_:H_2_O_2_:ascorbic acid = 1:1:4:20 and in the Cu(II):H_2_N‐GTHSAKHA‐NH_2_:H_2_O_2_ = 1:1:4 during 60 min oxidation is presented in Figure S7. The retention time of the peptide is 12.6 min. In the Cu(II):L:H_2_O_2_ = 1:1:4 system, only one product was formed in small quantity with a retention time of 13.6 min. In contrast, in the Cu(II):L:H_2_O_2_:ascorbic acid = 1:1:4:20 system, two products were formed, and the extent of oxidation was higher (Figure S7). Less than 10% of the peptide remained unchanged after 1 hour of oxidation. The products were identified by LC–MS measurements. A doubly and a singly oxidized product were observed at m/z 420.207 (calculated value 420.210) and at m/z 412.211 (calculated value 412.212) at 13.2 and 13.6 min, respectively (Figure S8). Since the intensity of the peaks was relatively low for further analysis, the products were isolated using a semipreparative column. The fractions were analyzed by MS and MS/MS methods. Different collision energy levels were applied (5, 10, 15, and 20 eV), of which 15 eV proved to be optimal. The MS/MS spectrum of the singly oxidized, doubly charged peptide [M + O + 2H]^2+^ is presented in Figure 3. According to the inset scheme in the upper‐left corner, peptide fragment ions are indicated by a, b, or c if the charge is retained on the N‐terminus and by x, y, or z if the charge is maintained on the C‐terminus. The subscript indicates the number of amino acid residues in the fragment [32]. The complementary ions are marked with the same color. It can be seen that the C‐terminal fragments contain the additional oxygen atom. The oxidation of the C‐terminal histidine is unambiguously proved by the presence of [y_2_ + O]^+^ and [z_2_ + O]^+^ with 242.125 m/z and 225.096 m/z (calculated values 242.125 and 225.098 m/z), respectively. Each of these fragments contains only one oxidation‐sensitive amino acid, histidine, in addition to alanine, which is resistant to oxidation. They differ solely in the position of peptide bond cleavage. Their corresponding N‐terminal pairs [b_6_]^2+^ and [c_6_]^2+^ with 291.656 m/z and 300.167 m/z (calculated values 291.656 and 300.167), are also present in the spectra without any additional oxygen atom. This indicates that the N‐terminal histidine is not oxidized.

**FIGURE 3:**
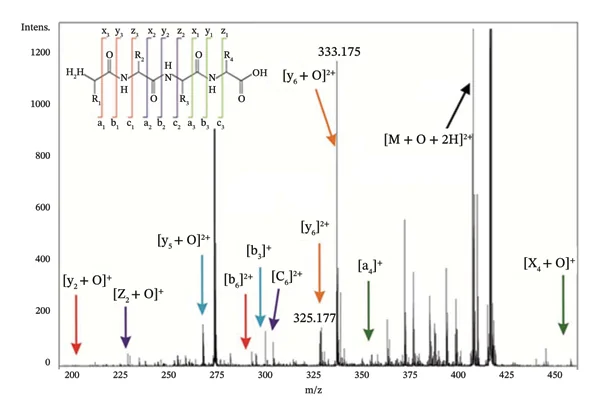
MS/MS spectrum of the singly oxidized products of H_2_N‐GTHSAKHA‐NH_2_ formed in the Cu(II):H_2_N‐GTHSAKHA‐NH_2_:H_2_O_2_:ascorbic acid = 1:1:4:20 system (inset scheme in the upper‐left corner: Peptide fragmentation notation using the scheme of Roepstorff and Fohlman for a tetrapeptide [32]).

[y_5_ + O]^2+^ with 265.145 m/z (calculated value 264.144) and [x_4_ + O]^+^ with 468.239 m/z (calculated value 468.239) and their pairs; [b_3_]^+^ with 296.134 (calculated value 296.135) and [a_4_]^+^ with 356.182 m/z (calculated value 356.180 m/z) also support the oxidation of the C‐terminal histidine, while the N‐terminal part of the peptide is not oxidized at all.

The above statements are also further supported by the [y_6_ + O]^2+^ with 333.175 m/z (calculated value 333.178), [b_5_]^+^ with 454.199 m/z (calculated value 454.204), and [b_4_]^+^ and 383.166 m/z (calculated value 383.167 m/z) ions in addition to the ion pairs mentioned earlier.

The effect of small molecules on the oxidation of the peptide was also investigated. The Cu(II)‐ion‐catalyzed oxidation of the H_2_N‐GTHSAKHA model peptide was studied by HPLC.

The comparison of the amount of the unoxidized peptide in the different systems after 1 h oxidation is presented in Figure 4. In the case of Cu(II):H_2_O_2_ system approximately 60% of the peptide remains in the solution after 1 h of oxidation. The addition of Salpyran prevents the oxidation process; more than 90% of the ligand remains unchanged. No main oxidation product was observed, and the formation of the 2‐oxo‐histidine derivative was not detected in this system, instead, the degradation of the peptide chain occurred, but to a small extent. Salpyran hinders the oxidation of the peptide by protecting the Cu(II) ions through the 2N, 2O coordination mode, inhibiting the ROS formation from the Cu(II)/H_2_O_2_ system.

**FIGURE 4:**
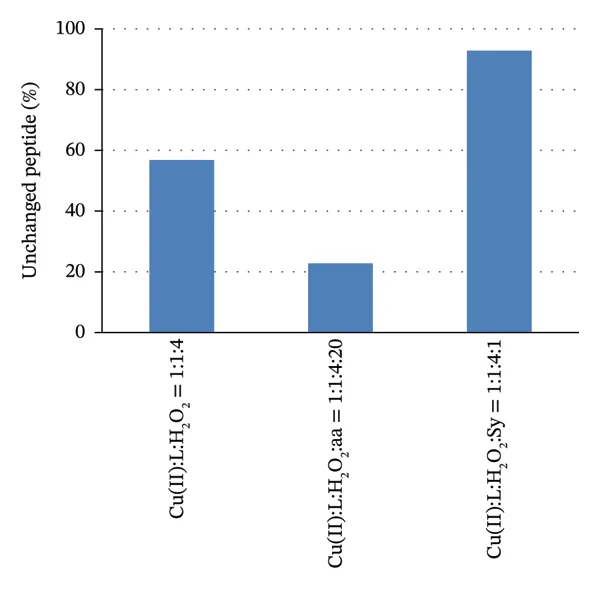
The amount of the unchanged peptide after 1 h oxidation in different systems. Abbreviations: aa: ascorbic acid, Sy: Salpyran.

However, when ascorbic acid was added to the system, the 2‐oxo‐histidine derivative was formed. Moreover, the presence of ascorbic acid increases the extent of peptide oxidation.

### 3.5. Ascorbate Consumption

Ascorbic acid consumption is often associated with the production of ROS. As a result, the characteristic band for ascorbic acid at 265 nm decreases. The Cu(II):H_2_N‐GTHSAKHA‐NH_2_ complex does not react effectively with ascorbic acid, as only a small decrease in absorbance is observed. Figure 5 shows that adding H_2_O_2_ to the system slightly accelerates this reaction. However, compared to uncomplexed Cu(II) ions, the reaction is still much slower.

**FIGURE 5:**
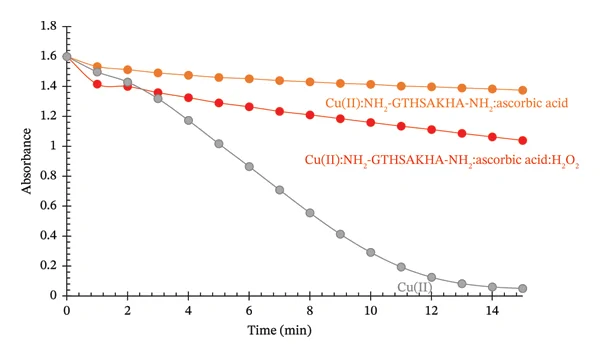
Ascorbate consumption by the Cu(II):H_2_N‐GTHSAKHA‐NH_2_ complex with/without H_2_O_2_ in PBS buffer at pH 7.4.

To confirm the production of •OH, an additional experiment was performed with R6G as a detector of this free radical. The detector shows a maximum absorption band at 526 nm. If a •OH is formed, it reacts with R6G and causes its degradation. The control reactions are almost at the same level as the reaction with Cu(II):H_2_N‐GTHSAKHA‐NH_2_ complex in the presence of H_2_O_2_, suggesting that the free radical is not produced at all in this system or is at a very low level (Figure 6).

**FIGURE 6:**
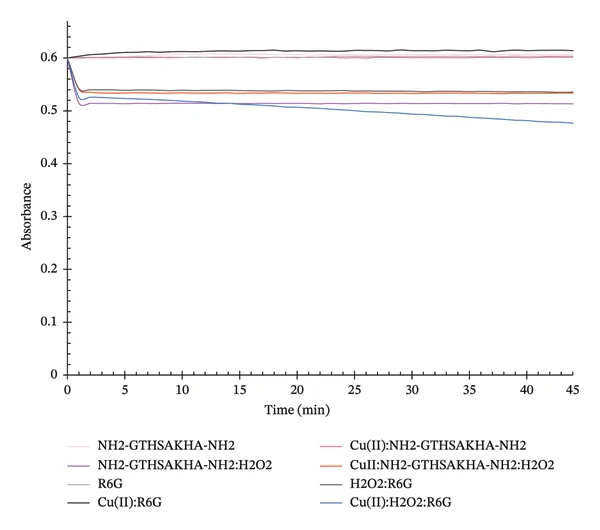
Absorbance versus time dependence for R6G with the addition of appropriate substances and with/without H_2_O_2_.

In addition, gel electrophoresis was also used to confirm the low activity of the Cu(II):H_2_N‐GTHSAKHA‐NH_2_ complex in the presence of H_2_O_2_ for ROS production. As can be seen in the electropherogram (Figure 7, Lane 4), the tested complex without the addition of hydrogen peroxide does not cause the generation of ROS that would damage DNA. These results are consistent with those for the control reactions (Figure 8) and the plasmid itself (Figure 7, Lane 1). The addition of H_2_O_2_ to the complex causes only minor DNA damage (Figure 7, Lane 5). The formation of 46% of the circular form of DNA is observed (compared to 22% for the control, Figure 7, Lane 5 compared to Lane 1). This is consistent with our previous results obtained using the UV–vis method, which confirmed that the Cu(II):H_2_N‐GTHSAKHA‐NH_2_ complex in the presence of H_2_O_2_ produces ROS at a very low level.

**FIGURE 7:**
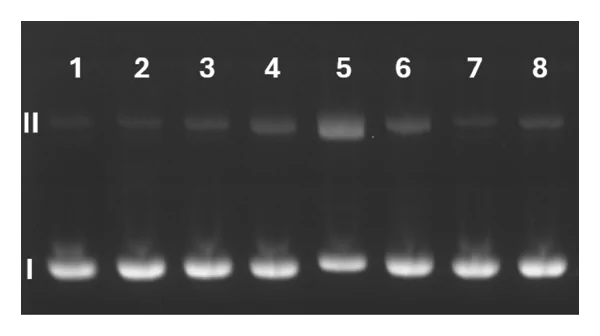
Agarose gel electrophoresis of pBR322 plasmid cleavage by H_2_N‐GTHSAKHA‐NH_2_ peptide (L), Cu(II):H_2_N‐GTHSAKHA‐NH_2_ complex (CuL) with/without H_2_O_2_ (50 μM) at pH 7.4. Lanes: 1: plasmid; 2: plasmid + L; 3: plasmid + L + H_2_O_2_; 4: plasmid + CuL; 5: plasmid + CuL + H_2_O_2_; 6: plasmid + CuL + H_2_O_2_ + 0.14 M DMSO; 7: plasmid + CuL + H_2_O_2_ + 40 mM NaN_3_; 8: plasmid + CuL + H_2_O_2_ + 8 mM KI.

**FIGURE 8:**
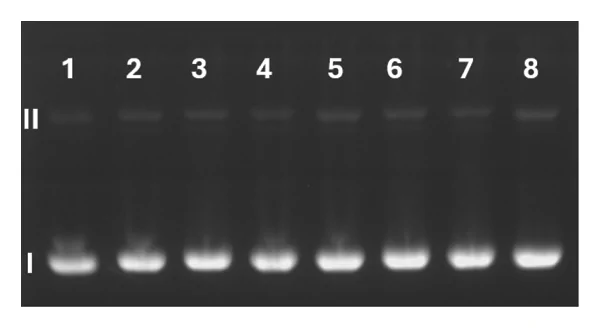
Agarose gel electrophoresis of pBR322 plasmid with/without H_2_O_2_ (50 μM) and scavengers at pH 7.4. Lanes: 1: plasmid; 2: plasmid + H_2_O_2_; 3: plasmid + 0.14 M DMSO; 4: plasmid + 40 mM NaN_3_; 5: plasmid + 8 mM KI; 6: plasmid + H_2_O_2_ + 0.14 M DMSO; 7: plasmid + H_2_O_2_ + 40 mM NaN_3_; 8: plasmid + H_2_O_2_ + 8 mM KI.

The situation changes completely after adding ascorbic acid to the Cu(II):H_2_N‐GTHSAKHA‐NH_2_: H_2_O_2_ system. Not only Form II of DNA (79%), but also Form III of DNA (21%) is formed (Figures 9 and 10, Lane 5). This means that the Cu(II):H_2_N‐GTHSAKHA‐NH_2_:ascorbic acid:H_2_O_2_ system produces ROS, which are aggressive to DNA and cause double‐strand DNA breaks (Figure 9, Lane 5). In this case, the native form of DNA is no longer observed. To check what kind of ROS is formed in solution and has a negative effect on DNA, specific ROS scavengers (NaN_3_ for ¹O₂, DMSO for ^•^OH and KI for O_2_^•−^) were used. Control reactions for the DNA in the presence of various scavengers and a mixture of H_2_O_2_ and Asc are shown in Figure S9. The addition of quenchers to the Cu(II):H_2_N‐GTHSAKHA‐NH_2_:ascorbic acid:H_2_O_2_ system allows for the uptake of ROS, which protects DNA from some damage (Figure 9, Lanes 6–8). In Lane 6, where DMSO was added to the complex containing a mixture of ascorbic acid and hydrogen peroxide, the existence of three forms of DNA was observed (Form I—25%, Form II—63%, and Form III—12%, Figures 9 and 10). Comparing this lane with Lane 5, the presence of a superhelical form of DNA, which is not present in Lane 5 can be observed. In the case of the reaction of NaN_3_ and KI in Cu(II):H_2_N‐GTHSAKHA‐NH_2_:ascorbic acid:H_2_O_2_ system, the quenching of ROS can be observed, which led to the formation of Form III of DNA (Figure 9, compare Lanes 7 and 8 with Lane 5). Moreover, in both cases the native form of DNA is also observed (Figure 9, Lanes 7 and 8). The results obtained by gel electrophoresis confirmed that ¹O₂, ^•^OH and O_2_^•-^ are generated in the Cu(II):H_2_N‐GTHSAKHA‐NH_2_:H_2_O_2_ system upon the addition of ascorbic acid. We have demonstrated that the addition of ascorbic acid to the complex in the presence of H_2_O_2_ leads to significantly enhanced oxidation of the histidyl residue and the increased formation of 2‐oxo‐histidine compared to the reaction in the absence of ascorbic acid. This effect can be explained by the substantially higher production of ROS in the Cu(II):H_2_N‐GTHSAKHA‐NH_2_:ascorbic acid:H_2_O_2_ system.

**FIGURE 9:**
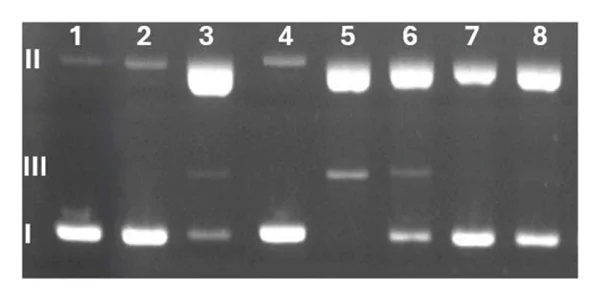
Agarose gel electrophoresis of pBR322 plasmid cleavage by H_2_N‐GTHSAKHA‐NH_2_ peptide (L), Cu(II):H_2_N‐GTHSAKHA‐NH_2_ complex (CuL) with/without ascorbic acid + H_2_O_2_ (50 μM) at pH 7.4. Lanes: 1: plasmid; 2: plasmid + L; 3: plasmid + L + ascorbic acid + H_2_O_2_; 4: plasmid + CuL; 5: plasmid + CuL + ascorbic acid + H_2_O_2_; 6: plasmid + CuL + ascorbic acid + H_2_O_2_ + 0.14 M DMSO; 7: plasmid + CuL + ascorbic acid + H_2_O_2_ + 40 mM NaN_3_; 8: plasmid + CuL + ascorbic acid + H_2_O_2_ + 8 mM KI.

**FIGURE 10:**
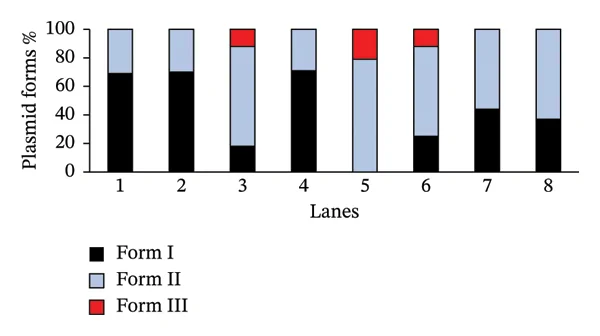
Densitometric analysis of the electropherogram shown in Figure 9.

## 4. Conclusions

The complex formation process of the studied peptide is similar to that of its methionine‐containing analog. Mono and dinuclear species are formed with the same stoichiometry with copper(II) and nickel(II) ions. In the case of zinc(II) ions, the studied peptide is also able to bind a second zinc ion, unlike the H_2_N‐GTHSMKHM‐NH_2_ peptide. The coordination begins at the N‐terminal site of the peptides, and the albumin‐like coordination is preferred for Cu(II), Ni(II), and Zn(II) ions with both peptides. The methionine residues have no significant contribution to the coordination of the first metal ion. However, the comparison of the stability constants does not provide any unambiguous evidence that the methionine residues act only as a bulky chain or have a stability‐enhancing effect on the coordination of the second metal ion. The same coordination mode exists in the mixed metal ion species formed in each ternary system. The metal ion binding preference is evident from these measurements. Copper(II) ions occupy the N‐terminal binding sites in each system; nickel(II) ions cannot compete with copper(II) for these sites, while zinc(II) ions bind only to the remaining free C‐terminal site of the peptide. Neither Ni(II) ions nor Zn(II) ions are able to alter the distribution of copper(II) ions among the available binding sites, in contrast to amyloid‐β [33, 34] or Hu–PrP [35] peptide fragments.

While the N‐terminal histidine is the preferred binding site, oxidation occurs at the C‐terminal site of the peptide. The oxidation of the second histidine residue occurs, and the 2‐oxo histidine derivative is formed by the Cu(II)/H_2_O_2_ system. However, the salan‐based Salpyran ligand protects the histidine residue from oxidation, whereas ascorbic acid promotes the formation of the 2‐oxo histidine derivative and increases the extent of oxidation. This may be related to ROS generation by the complex.

The ascorbate consumption experiment shows that the Cu(II)–H_2_N‐GTHSAKHA‐NH_2_ complex, in the presence of ascorbate alone, does not efficiently promote ascorbate oxidation, indicating that only a limited amount of ROS is generated under these conditions. A similar trend is observed in the R6G degradation experiment, in which H_2_O_2_ was added to the Cu(II)–H_2_N‐GTHSAKHA‐NH_2_ complex. Only a negligible decrease in absorbance at 526 nm was detected, suggesting that the concentration of •OH in the system is low. These results indicate that neither ascorbate nor H_2_O_2_, when applied separately, leads to significant hydroxyl radical production in the presence of the complex. This suggests that the complex stabilizes Cu(II) and limits ROS generation under single‐reducing agent conditions. However, as indicated by the ascorbate consumption assay, the simultaneous presence of ascorbate and H_2_O_2_ promotes more efficient reduction of Cu(II) to Cu(I), which in turn facilitates ROS generation through Cu(I)‐mediated reactions with H_2_O_2_.

In addition, we demonstrated that not only the peptide is oxidized by the formed ROS, but the ROS can also damage DNA.

## Funding

No funding was received for this manuscript.

## Conflicts of Interest

The authors declare no conflicts of interest.

## Supporting Information

Additional supporting information can be found online in the Supporting Information section.

## Supporting information


**Supporting Information** Supporting Information (SI) is available: Table S1: Measured spectroscopic data in the ternary systems. Figure S1: Species distribution of the complexes formed in the copper(II)–H_2_N‐GTHSAKHA‐NH_2_ systems as a function of pH. (a) c_Cu(II)_ = *c*_*L*_ = 1 · 10^−3^ M, (b) c_Cu(II)_ = 2 · 10^−3^ M, *c*_*L*_ = 1 · 10^−3^ M. Figure S2: (a) UV–vis and (b) CD spectra of the Cu(II):H_2_N‐GTHSAKHA‐NH_2_ system at 1:1 metal‐to‐ligand ratio at different pH values (*c*_*L*_ = 1*·*10^*−*3^ M). Figure S3: Experimental and simulated EPR spectra recorded for the Cu(II)‒NH_2_‐GTHSAKHA‐NH_2_ at concentration *c*_L_ = c_Cu_ = 1 mM (a) in aqueous solution at 295K. EPR parameters *g*_0_ = 2.097, *A*_0_ = 88 G were used for the simulation (b) at 77K. EPR parameters g_┴_ = 2.042, g_‖_ = 21,831, A_┴_ = 20 G and A_‖_ = 199 G were used for the simulation. Figure S4: (a) UV–vis and (b) CD spectra of the Cu(II):H_2_N‐GTHSAKHA‐NH_2_ system at 2:1 metal to ligand ratio at different pH values (*c*_*L*_ = 1*·*10^*−*3^ M). Figure S5: CD spectra of the Ni(II):H_2_N‐GTHSAKHA‐NH_2_ system at 2:1 metal to ligand ratio at different pH values (*c*_*L*_ = 1*·*10^*−*3^ M). Figure S6: UV–vis spectra of the Cu(II):Zn(II):H_2_N‐GTHSAKHA‐NH_2_ = 1:1:1 (a) and Ni(II):Zn(II):H_2_N‐GTHSAKHA‐NH_2_ = 1:1:1 (b) systems (*c*_*L*_ = 1*·*10^*−*3^ M). Figure S7: HPLC chromatograms of the peptide and the products formed in the Cu(II):H_2_N‐GTHSAKHA‐NH_2_:H_2_O_2_ = 1:1:4 and in the Cu(II): Cu(II):H_2_N‐GTHSAKHA‐NH_2_:H_2_O_2_:ascorbic acid = 1:1:4:20 systems during 60 min oxidation. Figure S8: MS spectrum of the Cu(II):H_2_N‐GTHSAKHA‐NH_2_:H_2_O_2_:ascorbic acid = 1:1:4:20 system after 1 hour oxidation. Figure S9: Agarose gel electrophoresis of pBR322 plasmid with ascorbic acid + H_2_O_2_ (50 μM) and scavengers at pH 7.4. Lanes: 1: plasmid; 2: plasmid + ascorbic acid + H_2_O_2_; 3: plasmid + ascorbic acid + H_2_O_2_ + 0.14 M DMSO; 4: plasmid + ascorbic acid + H_2_O_2_ + 40 mM NaN_3_; 5: plasmid + ascorbic acid + H_2_O_2_ + 8 mM KI.

## Data Availability

The data that support the findings of this study are available on request from the corresponding author. The data are not publicly available due to privacy or ethical restrictions.
