# Supplementary material for: Coordination and Metal Ion–Catalyzed Oxidation of a Mutated Prion Protein Fragment
Source: Bioinorg Chem Appl. 2026 Oct 21;2026:8800140. doi: 10.1155/bca/8800140 (PMC13687791; doi:10.1155/bca/8800140)
Supplement: Supplementary file 1 [file BCA-2026-8800140-s001.pdf]

SUPPORTING INFORMATION

Coordination and Metal Ion-Catalyzed Oxidation of a Mutated Prion Protein Fragment

Antal Galvácsi[a], Roland Császár[a], Lajos Nagy[b], Monika Lesiów[c*], Nóra V. May[d], Csilla Kállay[a*]

[a]*Department of Inorganic and Analytical Chemistry, University of Debrecen, H-4032 Debrecen, Hungary*

[b]*Department of Applied Chemistry, University of Debrecen, H-4032 Debrecen, Hungary*

[c]*Faculty of Chemistry, University of Wrocław, F. Joliot-Curie 14, 50-383 Wrocław, Poland*

[d]*Centre for Structural Science, Research Centre for Natural Sciences, Hungarian Research Network (HUN-REN), Magyar tudósok körútja 2, H-1117 Budapest, Hungary*

corresponding author: kallay.csilla@science.unideb.hu

Table S1: Measured spectroscopic data in the ternary systems

	Method	Cu(II)-Ni(II)-NH_2-GTHSAKHA-NH_2	Cu(II)-Zn(II)-NH_2-GTHSAKHA-NH_2	Ni(II)-Zn(II)-NH_2-GTHSAKHA-NH_2
below pH 8	UV–Vis (λ_{max}, nm)	532(120)	532	427
	CD ($\lambda_{max}/\Delta\varepsilon$, nm/ $dm^3\ mol^{-1}\ cm^{-1}$)	571/−0.99, 488/+0.45, 312/+1.96	571/−0.92, 489/+0.53, 312/+1.86	481/−2.53, 413/+1.74
around pH 11	UV–Vis (λ_{max}, nm)	*	532	480 (sh), 427
	CD ($\lambda_{max}/\Delta\varepsilon$, nm/ $dm^3\ mol^{-1}\ cm^{-1}$)	589/−0.59, 504/+2.89, 312/+1.87	571/−0.87, 494/+0.66, 313/+1.63	481/−2.54, 413/+1.74

* The square planar band of Ni(II) ions is combined with the d-d transition of Cu(II) ions

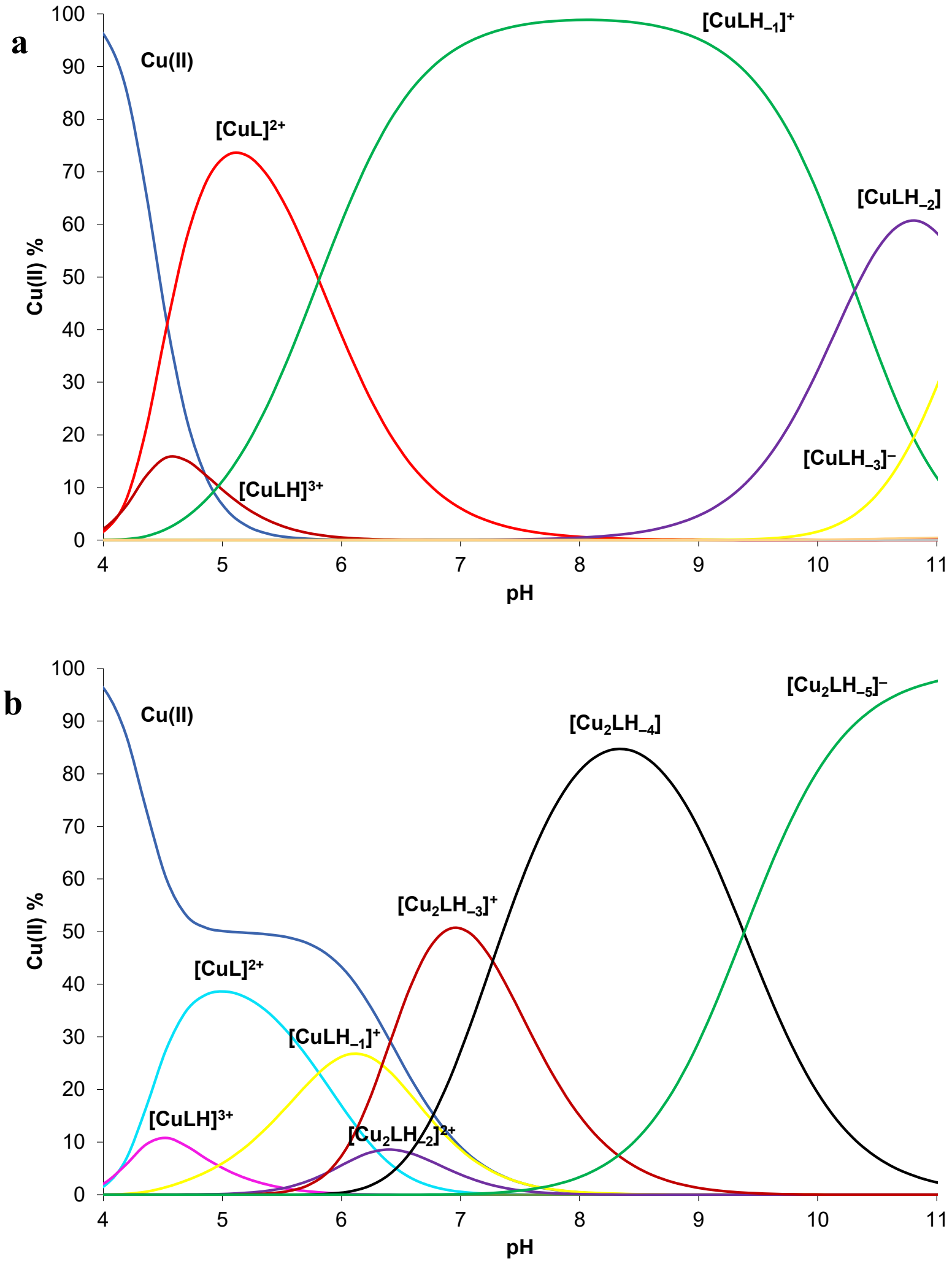

Figure S1: Species distribution of the complexes formed in the copper(II)–H_2N-GTHSAKHA-NH_2 systems as a function of pH. (a) $c_{Cu(II)} = c_L = 1 \cdot 10^{-3}$ M, (b) $c_{Cu(II)} = 2 \cdot 10^{-3}$ M, $c_L = 1 \cdot 10^{-3}$ M.

a

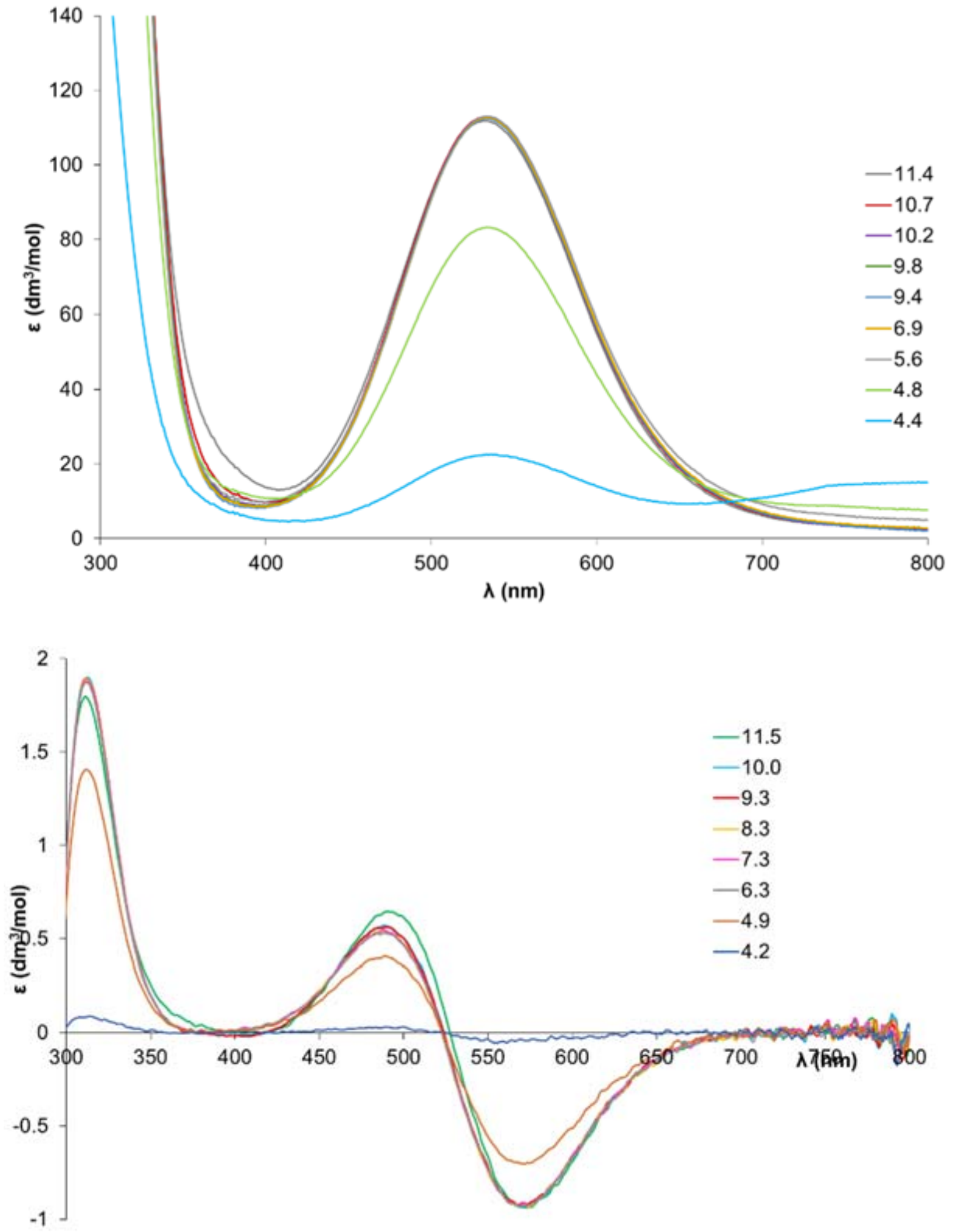

b

Figure S2: (a) UV-Vis and (b) CD spectra of the Cu(II):H_2N-GTHSAKHA-NH_2 system at 1:1 metal to ligand ratio at different pH values ($c_L = 1 \cdot 10^{-3}$ M).

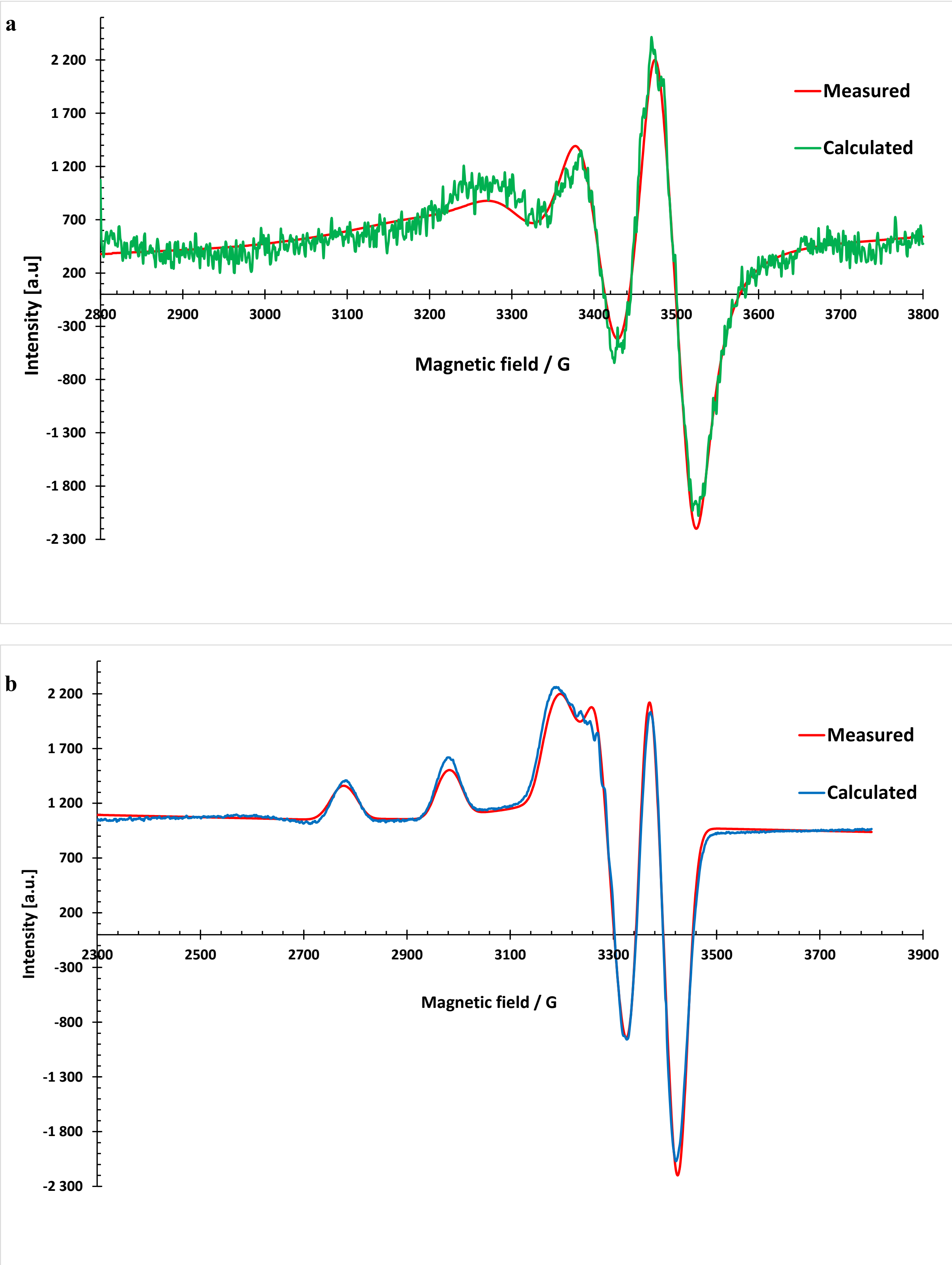

Figure S3: Experimental and simulated EPR spectra recorded for the Cu(II)–NH_2-GTHSAKHA-NH_2 at concentration $c_L = c_{Cu}$ = 1mM (a) in aqueous solution at 295K. EPR parameters g_0 = 2.097, A_0 = 88 G were used for the simulation (b) at 77K. EPR parameters $g_\perp$ = 2.042, $g_\parallel$ = 2,1831, $A_\perp$ = 20 G and $A_\parallel$ = 199 G were used for the simulation.

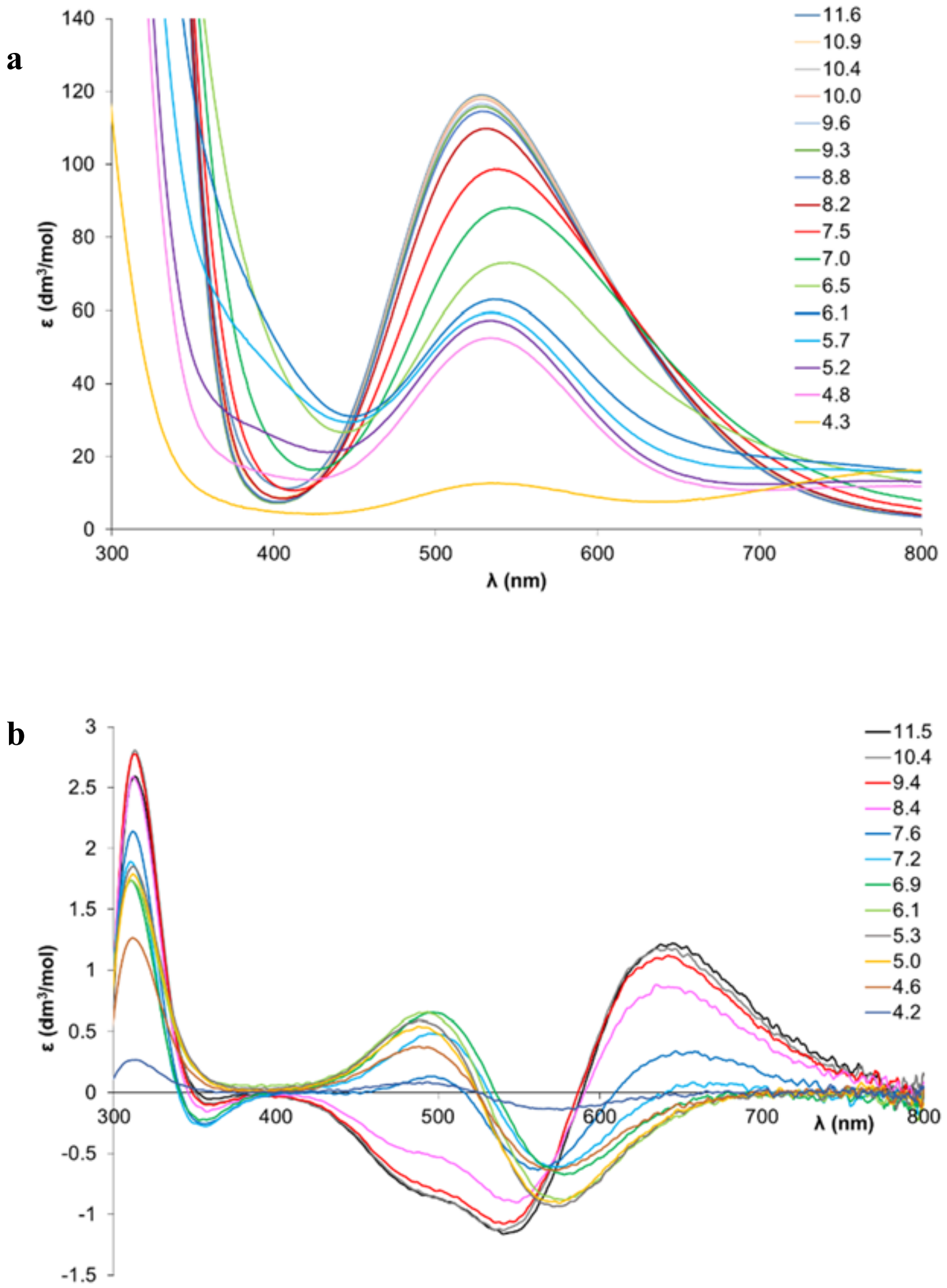

Figure S4: (a) UV-Vis and (b) CD spectra of the Cu(II):H_2N-GTHSAKHA-NH_2 system at 2:1 metal to ligand ratio at different pH values ($c_L = 1 \cdot 10^{-3}$ M).

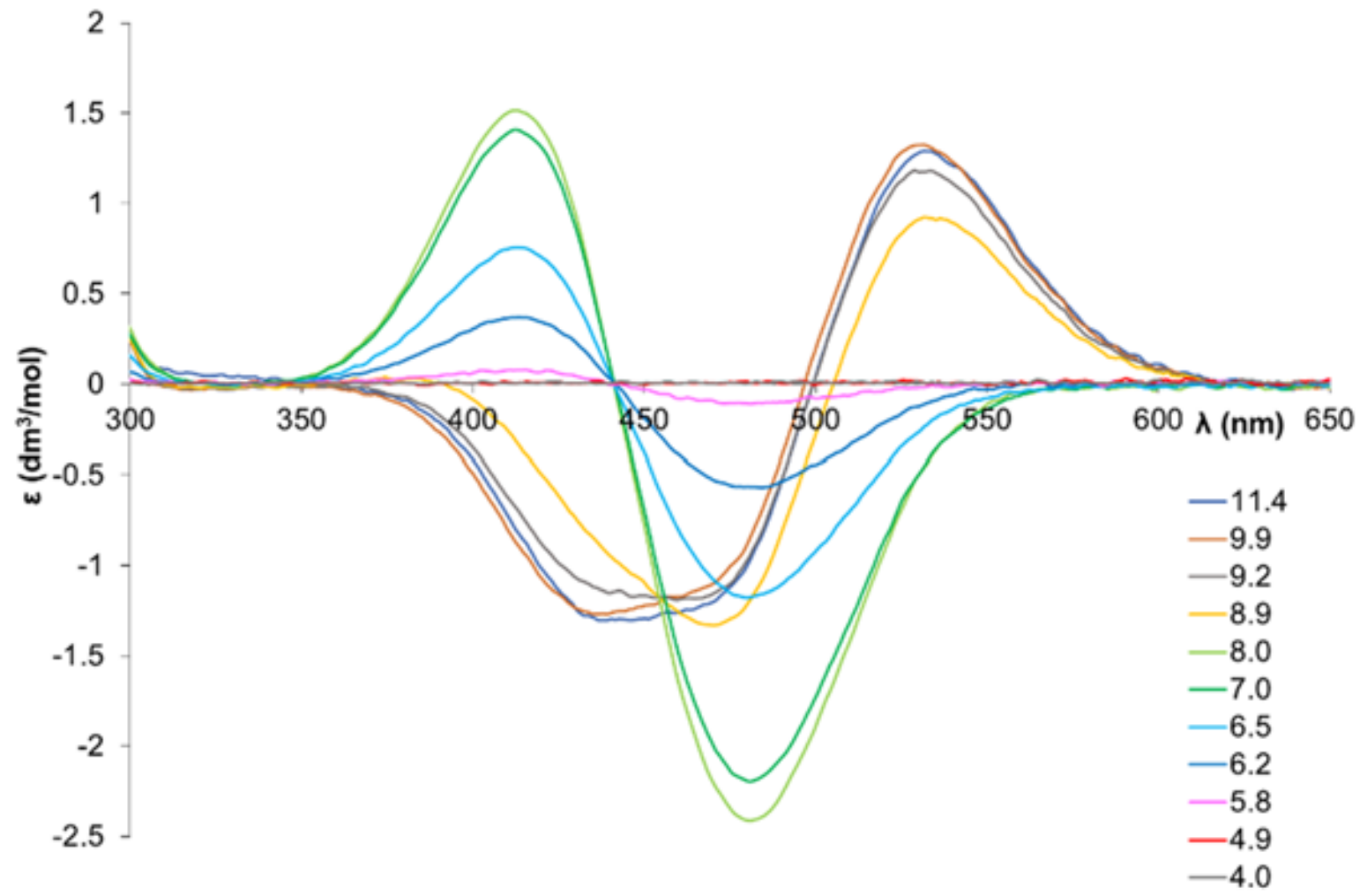

Figure S5: CD spectra of the Ni(II):H_2N-GTHSAKHA-NH_2 system at 2:1 metal to ligand ratio at different pH values ($c_L = 1{\cdot}10^{-3}$ M).

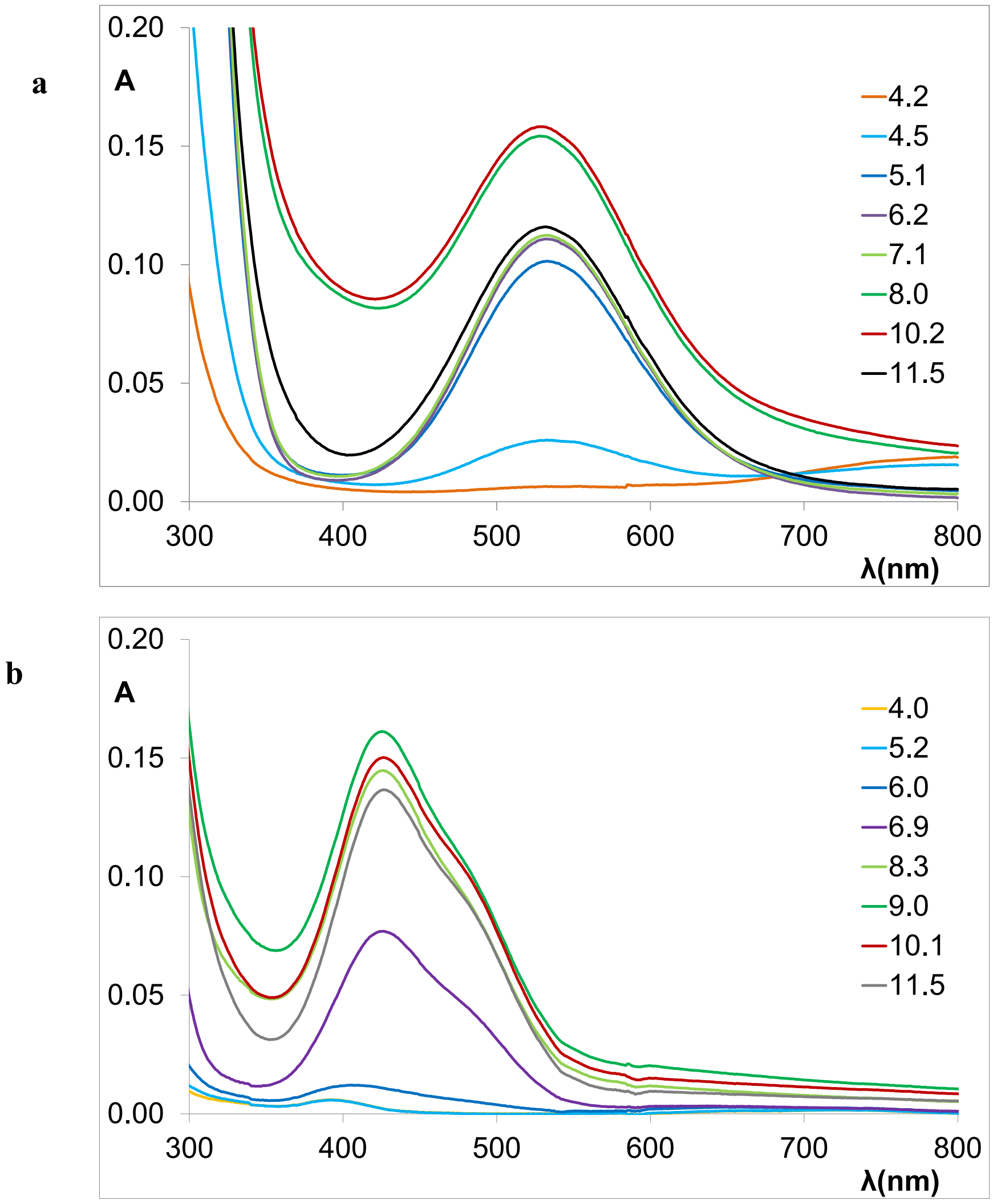

Figure S6: UV-Vis spectra of the Cu(II):Zn(II):H_2N-GTHSAKHA-NH_2 = 1:1:1 (a) and Ni(II):Zn(II):H_2N-GTHSAKHA-NH_2 = 1:1:1 (b) systems ($c_L = 1\cdot10^{-3}$ M).

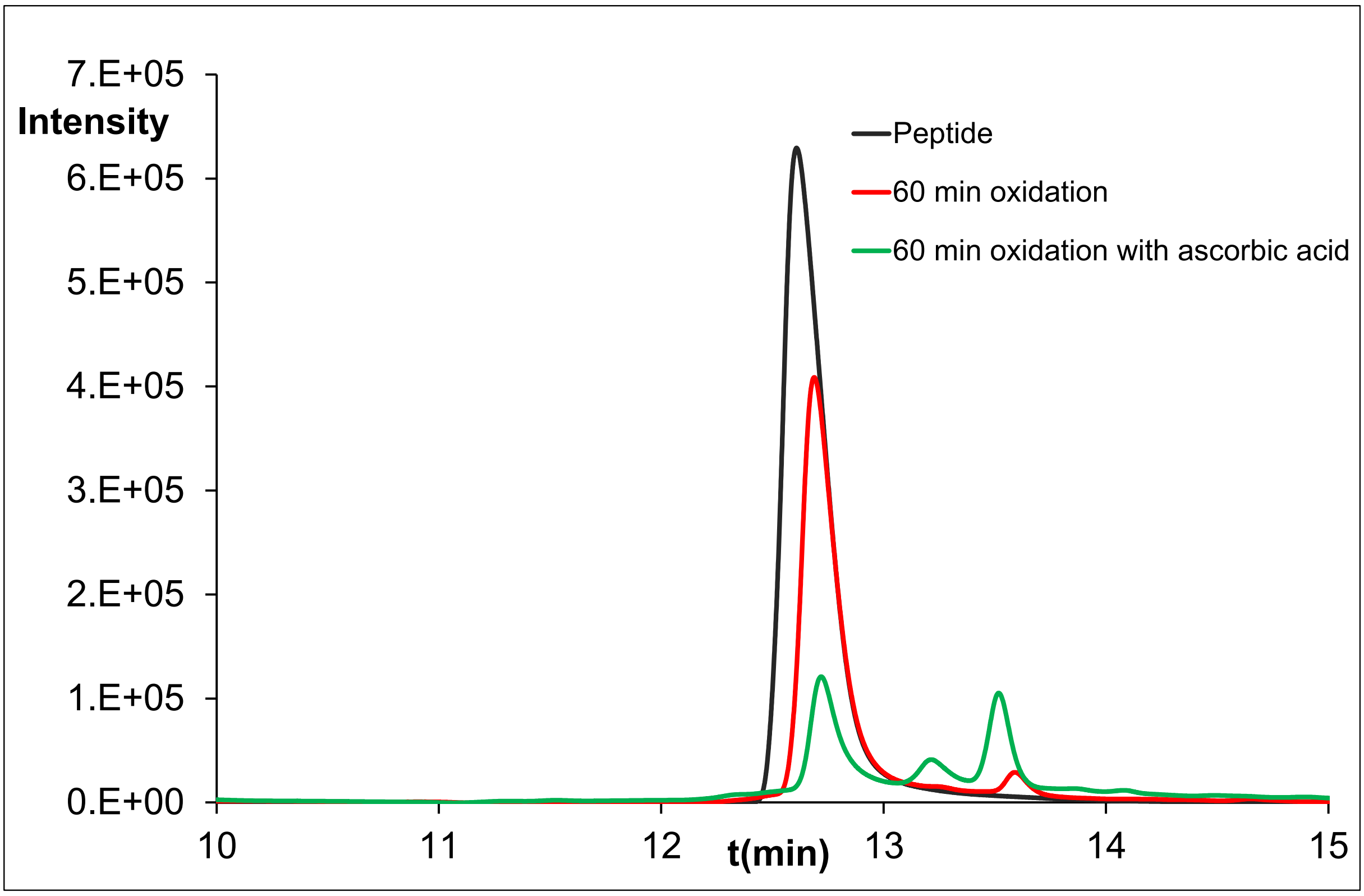

Figure S7: HPLC chromatograms of the peptide and the products formed in the Cu(II):H_2N-GTHSAKHA-NH_2:H_2O_2 = 1:1:4 and in the Cu(II): Cu(II):H_2N-GTHSAKHA-NH_2:H_2O_2:ascorbic acid = 1:1:4:20 systems during 60 minutes oxidation.

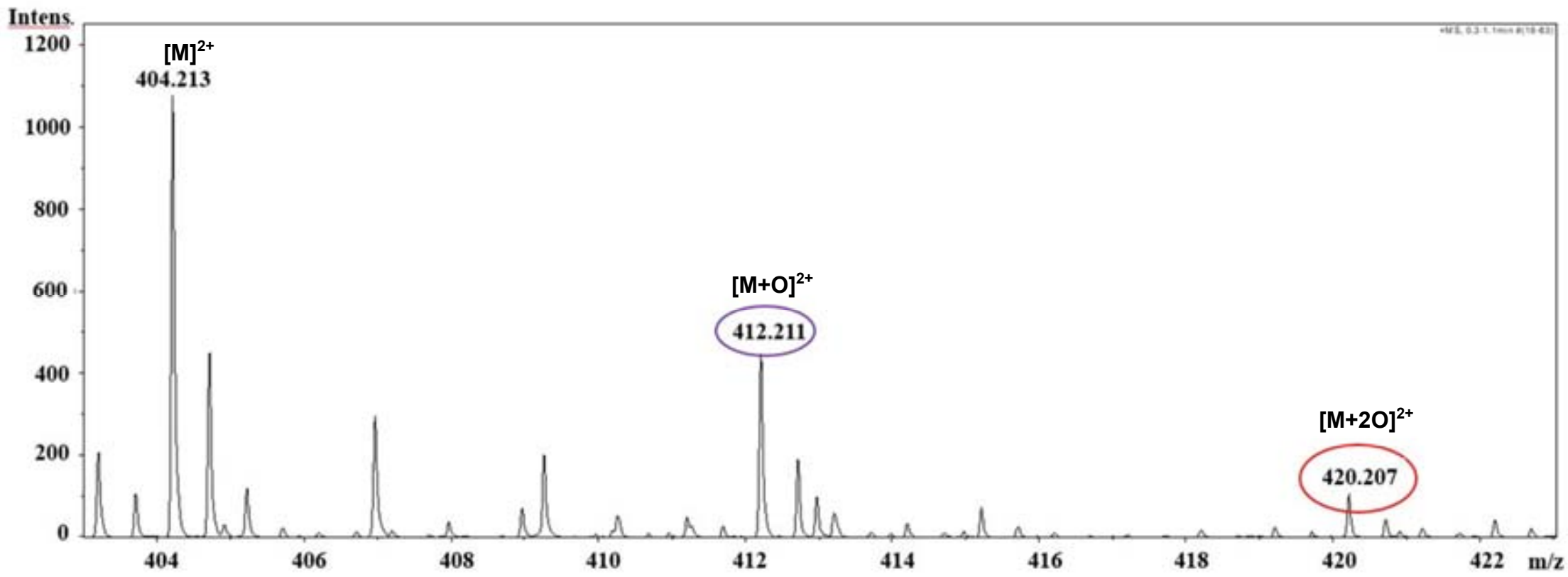

Figure S8: MS spectrum of the Cu(II):H_2N-GTHSAKHA-NH_2:H_2O_2:ascorbic acid = 1:1:4:20 system after one hour oxidation

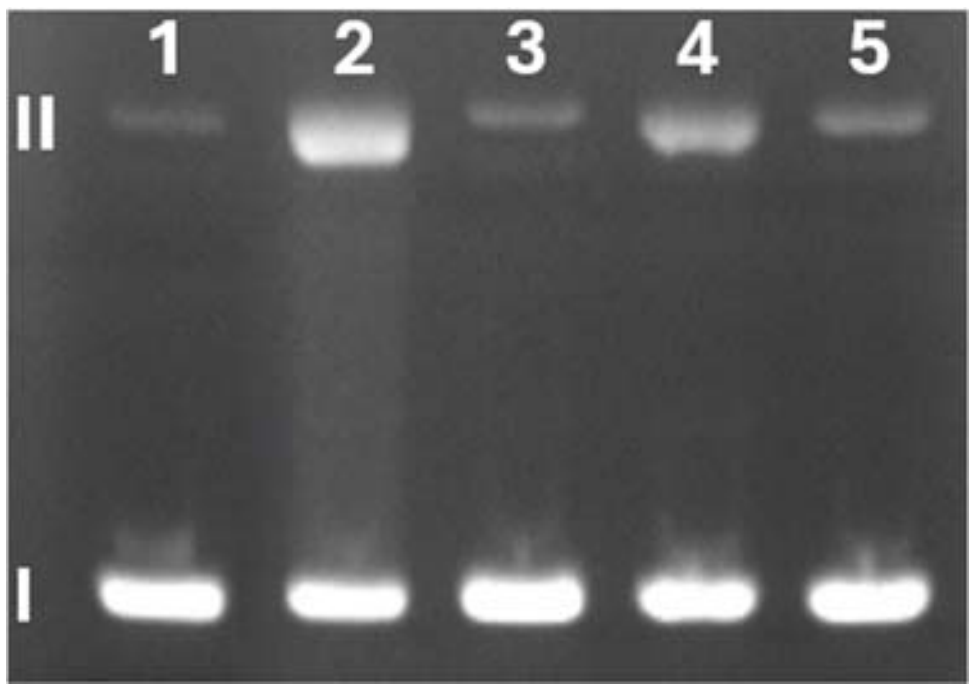

Figure S9: Agarose gel electrophoresis of pBR322 plasmid with ascorbic acid + H_2O_2 (50 μM) and scavengers at pH 7.4. Lanes: **1:** plasmid; **2:** plasmid + ascorbic acid + H_2O_2; **3:** plasmid + ascorbic acid + H_2O_2 + 0.14 M DMSO; **4:** plasmid + ascorbic acid + H_2O_2 + 40 mM NaN_3; **5:** plasmid + ascorbic acid + H_2O_2 + 8 mM KI.
